# Effects of Canine IL-12 on the Immune Response Against the Canine Parvovirus VP2 Protein

**DOI:** 10.3390/vaccines13070758

**Published:** 2025-07-16

**Authors:** Shiyan Wang, Wenjie Jiao, Dannan Zhao, Yuzhu Gong, Jingying Ni, Huawei Wu, Jige Du, Tuanjie Wang, Chunsheng Yin

**Affiliations:** 1 China Institute of Veterinary Drug Control, Beijing 100081, China; shiyanwang11@163.com (S.W.);; 2 College of Veterinary Medicine, Hunan Agricultural University, Changsha 410128, China

**Keywords:** canine parvovirus, VP2, IL-12, baculovirus expression system

## Abstract

**Background:** Canine parvovirus (CPV) is a highly pathogenic virus that predominantly affects puppies, with mortality rates exceeding 70%. Although commercial multivalent live attenuated vaccines (MLV) are widely employed, their efficacy is often compromised by maternal antibody interference. Consequently, the development of novel vaccines remains imperative for effective CPV control. **Methods:** Recombinant CPV VP2 protein (rVP2) and canine interlukine 12 protein (rcIL-12) were expressed using the Bac-to-Bac baculovirus expression system and the biological activity of these proteins was assessed through hemagglutination, Cell Counting Kit-8 (CCK8) and IFN-γ induction assays. The combined immunoenhancement effect of rVP2 and rcIL-12 protein was evaluated in puppies. **Results:** Both rVP2 and rcIL-12 were successfully expressed and purified, exhibiting confirmed antigenicity, immunogenicity, and bioactivity. Co-administration of rVP2 with rcIL-12 elicited higher neutralizing antibody titer (6–7 times higher), complete challenge protection efficiency (no clinical symptoms and tissue and organ lesions), fewer viral shedding (decreasing significantly 8-day post challenge) and superior viral blockade (lower viral load in the organism) compared to rVP2 alone. **Conclusions:** Our findings demonstrate that rVP2 co-administered with rcIL-12 induces robust protective immunity in puppies and significantly mitigated the inhibitory effects of maternal antibodies. This represents a promising strategy for enabling earlier vaccination in puppies and rational design of CPV subunit vaccines.

## 1. Introduction

Canine parvovirus (CPV) is a non-enveloped virus characterized by an icosahedral capsid (25 nm in diameter) encapsulating a linear single-stranded negative-sense DNA genome [1]. The viral particle exhibits icosahedral symmetry. CPV comprises two genotypes: CPV-1 (also known as canine minute virus), which is relatively rare and exhibits low pathogenicity, and CPV-2, the predominant pathogenic subtype. CPV-2 has further evolved into three antigenic variants (CPV-2a, -2b, and -2c). Notably, CPV-2 shares 99% genomic sequence homology with feline panleukopenia virus (FPV) [2], supporting the hypothesis of its evolutionary origin from FPV through host adaptation.

Vaccination remains the most effective measure to control clinical CPV infection and transmission among dogs. However, despite intensive global vaccination efforts, CPV remains a prevalent infectious disease with frequent breakthrough infections, largely attributed to improper vaccination protocols [3]. Early vaccination before 8 weeks of age is often hindered by maternal antibody interference, reducing protective immunity. Therefore, developing vaccines that overcome maternal antibodies and optimizing vaccination schedules are critical.

VP2 is the major capsid protein of CPV, containing key antigenic determinants, host range–defining sites, and the binding site for the host cell transferrin receptor [4]. As a principal capsid component, VP2 is commonly used in recombinant subunit vaccines. Multiple studies have demonstrated that VP2 expressed via a baculovirus system can self-assemble into virus-like particles (VLPs) resembling native structures, effectively inducing robust protective immunity in dogs [5,6].

IL-12, a member of the IL-12 cytokine family sharing the IL-12B subunit with IL-23, is the first identified heterodimeric cytokine produced by antigen-presenting cells in response to pathogen-associated molecular patterns [7]. IL-12 shows great potential as a vaccine adjuvant for infectious diseases, demonstrated by its ability to reduce eosinophil counts in patients with airway hyperresponsiveness [8], enhance T-cell responses against hepatitis B virus [9,10], and improve DNA vaccine immunogenicity [11]. Additionally, IL-12 serves as a mucosal adjuvant; for instance, intranasal immunization with a soluble hemagglutinin, neuraminidase, and IL-12 influenza vaccine significantly increased survival and reduced clinical symptoms in mice after viral challenge [12]. Oral administration of IL-12 liposomes has also been shown to elevate Th2 cytokine levels [13].

## 2. Materials and Methods

### 2.1. Molecular Cloning

The CPV VP2 gene was optimized and synthesized by Sangon Biotech (Shanghai, China), based on the CPV-2c VP2 Structural protein gene (GenBank: KY937650). The IL-12 gene was connected by the order of N-P40-P30-C and then optimized and synthesized by Sangon Biotech, based on the open reading frame region (ORF) of the canine IL-12 gene (GenBank: NC_051838). The two genes were amplified by primer pairs with Homology arms, and then recombinated with Linearized pFastBacHT A plasmid using the ClonExpress Ultra One Step Cloning Kit (Vazyme Biotech, Nanjing, China).

The two recombinant plasmids were transformed into *E. coli* DH5α competent cells following the instruction to amplify the plasmids. Positive colonies were selected by PCR and sequencing assays. The two recombinant plasmids were named pFastBacHTA-rVP2 and pFastBacHTA-rcIL12, respectively. To obtain purified plasmids, the FastPure Plasmid Mini Kit (Vazyme Biotech) was used for subsequent experiments.

### 2.2. Construction of Recombinant Baculovirus

The two recombinant plasmids were transformed into into *E. coli* DH10bac competent cells following the instructions to obtain recombinant bacmids. Positive colonies were selected by Blue-White Screening assay twice to insure no empty plasmids. PCR and sequencing assays were used in subsequent identification. Recombinant bacmids were purified by the PureLink HiPure Plasmid Midiprep Kit (Invitrogen, Carlsbad, CA, USA), and then 1 μg of bacmid DNA was transfected to Sf9 cells in a six-well cell plate (1 × 10^6^/well, Cell attachment) using ExpiFectamine Sf^TM^ Transfection Reagent (Invitrogen), referring to the manual instruction. Incubating in 27 °C for 72–96 h and then centrifugating at 500× *g* for 2 min, recombinant baculovirus were assembled and obtained. New generation baculovirus was amplified in suspension Sf9 cells (whose density was 1 × 10^6^/mL and cultured in 27 °C at 125 rpm/min).

### 2.3. Protein Expression and Purification

The titer of recombinant baculovirus was determined using the BacPAK Baculovirus Rapid Titer Kit (Takara Shuzo, Osaka, Japan). About a third generation of baculovirus, whose titer was greater than 1 × 10^8^ IFU/mL, was permitted to infect High Five cells to express protein. Suspension High Five cells (same culture condition as Sf9) were infected by recombinant baculovirus for multiplicity of infection (MOI) 5. Cells were centrifuged at 500× *g* for 2 min and collected after 60 h.

Collected cells were dissolved in LE buffer (100 mmol NaH_2_PO_4_, 10 mmol Tris-Cl, 8 M Urea, pH 8.5), flowed through High Affinity Ni-Charged Resin FF (Genscript, Nanjing, China), and then eluted using Elution Buffer (100 mmol NaH_2_PO_4_, 10 mmol Tris-Cl, 8 M Urea, 500 mmol imidazole, pH 8.5). Liquid of Eluent was performed dialysis in the storage buffer (100 mmol NaH_2_PO_4_, 10 mmol Tris-Cl, 1.5 M NaCl, 10% glycerin, pH 8.5).

### 2.4. SDS-PAGE and Western Blotting Analysis

Two proteins acquired from 2.3 were mixed with 4× loading buffer and boiled for 10 min to denaturate protein for SDS-PAGE. Treated proteins were loaded in SurePAGE™ protein preform glue (Genscript), which was run at 120 V for 45 min. The protein was transferred to a PVDF membrane at 200 V for 2 h, using Mini-PROTEAN Tetra Vertical Electrophoresis (Bio-Rad, Hercules, CA, USA). The rVP2 protein was detected by CPV monoclonal antibody (PL Laboratories, Beijing, China), and the rcIL-12 protein was detected by canine IL-12 antibody (Bio-techne, Minneapolis, MN, USA; 1:2000). Then the PVDF membranes were incubated in diluted second antibody connected with HRP flag (1:5000) and exposured in Amersham^TM^ Imager 600 (Cytiva, Uppsala, Sweden).

### 2.5. Evaluation of Biological Activity

The 1% red blood cell suspension was collected from a Guinea pig, washed for 3 times using PBS, and diluted by 0.9% NaCl. The purified rVP2 protein was mixed with 1% red blood cell suspension in 1:1 ratio and incubated in room temperature for 1 h.

To validate the bioactivity of rcIL-12 on promoting PBMC proliferation combined with IL-2, four groups were set up: a blank control group, rcIL-12 group, IL-2 group, and rcIL-12+IL-2 group. 50 μL of 100 μg/mL rcIL-12 protein, IL2 protein, rcIL-12+IL2 protein and 50 μL of 10% FBS were added to the canine peripheral blood mononuclear cells (PBMCs) wells, respectively. After 16 h of incubation, 10 μL of CCK8 reagent was added and reacted at 37 °C for 2 h. The absorbance was measured using BioTek Synergy HTX (BioTek, Winooski, VT, USA) at 450 nm.

To validate the bioactivity of rcIL-12 on inducing IFNγ in canine PBMC, rcIL-12 was serially diluted and co-cultured with PBMCs for 16 h. Total RNA was extracted from treated PBMCs, and the relative mRNA expression levels of IFN-γ were determined by Reverse transcription quantitative real-time PCR (qPCR) using the 2^−ΔΔCt^ method, with GAPDH as the internal reference gene. The IFN-γ primer pairs (F: 5′-GACGGTGGGTCTCTTTTCGT-3′, R: 5′-CCTGCAGATCGTTCACAGGA-3′) and GAPDH primer pairs (F: 5′-AGTTGTGGATCTGACCTGCC-3′, R: 5′-CAGGATGCCTTTGAGGGGTC-3′) were used. The concentration of IFN-γ in supernatants was measured using a canine IFN-γ ELISA kit (Jonlonbio, Shanghai, China).

### 2.6. Animals, Vaccination, and Challenge

F81 cells and CPV-2c (strain BJ1606) were obtained from the China Institute of Veterinary Drug Control (IVDC). Beagle puppies (20-day-old, non-immunized) were sourced from Beijing Hongrong’an Breeding Farm, with all animal experiments conducted under ABSL-2. Animal trials were approved by the Ethics Committee of IVDC and followed 3Rs principle to ensure animal welfare.

Beagle puppies (*n* = 6) were randomly allocated to two immunization groups (3/group): rVP2 group, rVP2+rcIL-12 group, with a control cohort (*n* = 3) of 2-month-old Beagles for challenge validation. All formulations were emulsified with 10% SEPPIC GEL 02 adjuvant and delivered via intramuscular injection. Serial serum collections were performed at 7-day intervals after immunization for 4 times, and virulent challenge were administered at day 39-post-primary immunization to evaluate protective efficacy. The experimental protocol is presented in Table 1.

### 2.7. Clinical Scores

Post-challenge, puppies were monitored daily for mental status, appetite, fecal consistency, and rectal temperature. Clinical scores were assigned to each group as shown in Table 2. Total scores (10 points) were calculated by summing all subscores, with higher values indicating worse clinical status.

### 2.8. Serum Neutralization Analysis

The virus neutralization assay was performed by heat-inactivating serum samples at 56 °C for 30 min, followed by preparation of two-fold serial dilutions. Equal volumes of diluted serum were mixed with CPV viral suspension standardized to 200 TCID_50_ and incubated at 37 °C for 1 h. The serum-virus mixtures were then transferred to F81 feline kidney cell monolayers in 96-well plates after removal of culture medium, with 100 μL per well. Following 4 days of incubation at 37 °C with 5% CO_2_, cells were fixed with 80% acetone and processed for indirect immunofluorescence assay using CPV-specific monoclonal primary antibody and FITC-conjugated secondary antibody. Neutralizing antibody titers were determined as the highest serum dilution demonstrating complete absence of fluorescent foci under epifluorescence microscopy with green excitation (compared to virus controls).

### 2.9. qPCR Quantitative Analysis of Rectal Swabs

Following challenge, anal swab samples were obtained from all canines at 48-h intervals. Swabs were vortexed in 500 μL PBS, and 300 μL aliquots were subjected to DNA extraction using the Stool DNA Kit (Omega Bio-Tek, Norcross, GA, USA). QPCR targeting the CPV gene was performed as described by Fu [14] using primer pairs (F: 5′-ACGTAGTGGACCTTGCACTG-3′, R: 5′-CCCTTACCTCTCCTGGCTCT-3′). Amplification condition: 95 °C for 30 s; 40 cycles of 95 °C (5 s) and 60 °C (34 s); with melt curve generation (95 °C, 15 s). The standard curve was Y = −3.389X + 38.792.

### 2.10. Histopathological Analysis

Dogs meeting euthanasia criteria (moribund, freshly deceased, or survivors at 14 days post-challenge) were humanely euthanized. Necropsy was performed to examine gross lesions in major organs (heart, liver, spleen, lungs, kidneys, and intestinal tract). Tissue samples spanning healthy and lesional margins were collected and immersion-fixed in 4% paraformaldehyde (PFA) for subsequent histological processing.

Hematoxylin and eosin (HE) staining was performed using an automated stainer (Leica ST5020, Nussloch, Germany) with the following protocol: (1) Deparaffinization and hydration through xylene and graded ethanol series (100% → 70%), (2) Nuclear staining with Mayer’s hematoxylin (5 min), (3) Differentiation in 1% acid alcohol (30 s), (4) Bluing in 0.2% ammonia water (1 min), (5) Cytoplasmic counterstaining with eosin Y (2 min). Stained sections were air-dried at room temperature (25 ± 2 °C) for 30 min before coverslipping with permanent mounting medium using an automated coverslipper.

The immunohistochemical staining was performed as follows: Tissue sections were deparaffinized in xylene and rehydrated through a graded ethanol series, followed by antigen retrieval in 0.05 M citrate buffer (pH 6.0) at 90 °C for 20 min. After cooling to room temperature and rinsing with PBS, endogenous peroxidase activity was blocked with 3% hydrogen peroxide in methanol for 30 min in the dark. Nonspecific binding was blocked with immunohistochemistry (IHC) blocking serum at 37 °C for 30 min. Sections were then incubated with mouse anti-CPV monoclonal antibody (1:200) at 4 °C overnight, followed by HRP-conjugated anti-mouse secondary antibody (1:500) at room temperature for 1 h. The immunoreaction was visualized using Vector VIP substrate (Vector Laboratories, Burlingame, CA, USA) for approximately 1 min, followed by counterstaining with hematoxylin for 45 s and bluing in running tap water for 10–15 min. Finally, sections were dehydrated through graded alcohols, cleared in xylene, and mounted with a 1:1 mixture of neutral resin and xylene.

### 2.11. Data Analysis

Statistical analysis was used in the data of this study. All the data are shown as mean ± SD and analyzed using Three-way ANOVA or One-way ANOVA statistical analysis for inter/intra-group comparisons. Graphical representations, including bar charts, line graphs, and Kaplan-Meier survival curves, were generated using GraphPad Prism 9 (GraphPad Software, San Diego, CA, USA).

## 3. Results

### 3.1. The Recombinant Baculovirus Was Successfully Constructed, Yielding Purified Recombinant rVP2 and rcIL-12 Proteins

**Figure 1:**
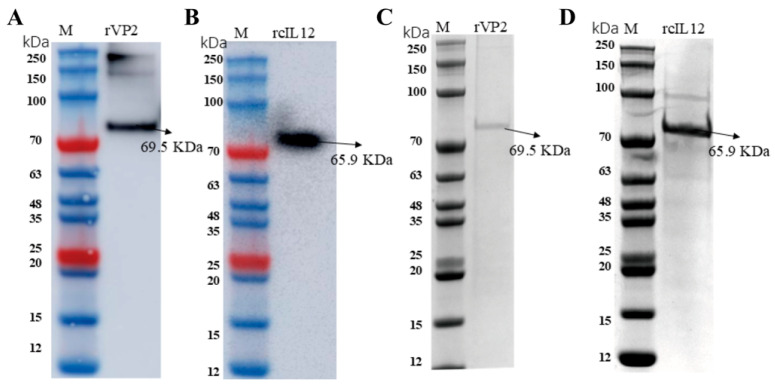
SDS-PAGE and Western blot analysis (**A**) WB probed with anti-CPV antibody. The Arrow marks the 69.5 kDa target band. (**B**) WB probed with canine IL-12 antibody. The Arrow marks the 65.9 kDa target band. (**C**) Coomassie blue-stained SDS-PAGE gel of rVP2. (**D**) Coomassie blue-stained SDS-PAGE gel of rcIL-12.

The two recombinant baculoviruses were generated using the Bac-to-Bac system (Thermo Fisher, Waltham, MA, USA). Target optimized genes (rVP2 and rcIL-12) were cloned into a pFastBacHT A vector, and recombinant bacmids were selected in *E. coli* DH10Bac and blue–white screening. All of these process were verified by PCR and sequencing. After transfection into Sf9 cells (1 × 10^6^ cells/well, 27 °C), viral propagation was monitored by syncytium formation after 72–96 h of culture. Protein expression was confirmed by WB using a specific antibody (Figure 1A,B), and purification was performed via Ni-NTA resin (Figure 1C,D). The two protein final preparation yielded 2–3 mg of >90% purity (as assessed by SDS-PAGE densitometry) per 50 mL culture volume.

### 3.2. The Two Recombinant Proteins Retained Biological Activities Comparable to Their Native Counterparts

**Figure 2:**
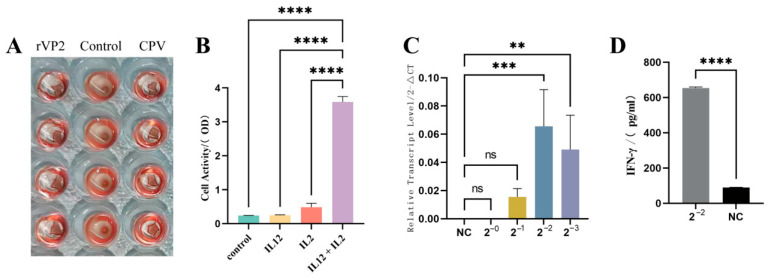
Bioactivity analysis of recombinant. (**A**) Hemagglutination assay. rVP2 protein were incubated with 0.1% guinea pig RBCs. PBS served as a control. (**B**) Cell proliferation assay. Canine PBMCs were treated with rcIL-12 and canine IL-2. Proliferation was measured by CCK-8 absorbance (450 nm). (**C**) IFN-γ induction. Canine PBMCs were stimulated with diluted rcIL-12. IFN-γ transcript level in PBMC was measured using RT-PCR. (**D**) Supernatants of (**C**) in 4-fold diluted were quantified by ELISA kit. Data represents mean ± S.D. (*n* = 5). ** indicates *p* < 0.01, *** indicates *p* < 0.001, **** indicates *p* < 0.0001 and ns indicates no significance.

Consistent with native CPV [15,16], the baculovirus-expressed rVP2 protein maintained hemagglutination functionality. In the HA assay, purified rVP2 (500 μg/mL) were incubated with 0.1% guinea pig RBCs in V-bottom microplates at room temperature for 1 h. The positive HA activity of rVP2 (Figure 2A) corroborates its structural integrity and receptor-binding capability.

Canine PBMCs cultured in 96-well plates (1 × 10^5^/well) were treated with rcIL-2 and recombinant rcIL-12 for 16 h, followed by 2 h incubation with CCK-8 reagent (37 °C). Meanwhile, the three control groups were set. OD450 measurements demonstrated that rcIL-12 synergized with IL-2 to significantly enhance PBMC proliferation (*p* < 0.0001 vs. IL-2 alone), which was consistent with established literature on IL-12 bioactivity [17,18]. Neither PBS nor rcIL-12 alone exhibited proliferative effects (Figure 2B).

The recombinant rcIL-12 protein was incubated with canine PBMCs for 16 h. Total RNA was extracted, reverse-transcribed into cDNA, and the relative transcriptional level of IFN-γ was quantified using the 2^−ΔΔCT^ method with GAPDH as the internal reference. Diluted rcIL-12 significantly upregulated IFN-γ transcription in PBMCs (*p* < 0.05) in a dose-dependent manner (Figure 2C). In contrast, the negative control and undiluted rcIL-12 group showed no detectable CT values in qPCR, indicating negligible IFN-γ transcription. Consistent with qPCR results, IFN-γ ELISA of culture supernatants from PBMCs treated with four-fold diluted rcIL-12 revealed a marked increase in IFN-γ secretion (*p* < 0.001; Figure 2D).

### 3.3. The Combined Immunization with rVP2 and rcIL-12 Demonstrated Superior Efficacy Compared to rVP2 Alone

**Figure 3:**
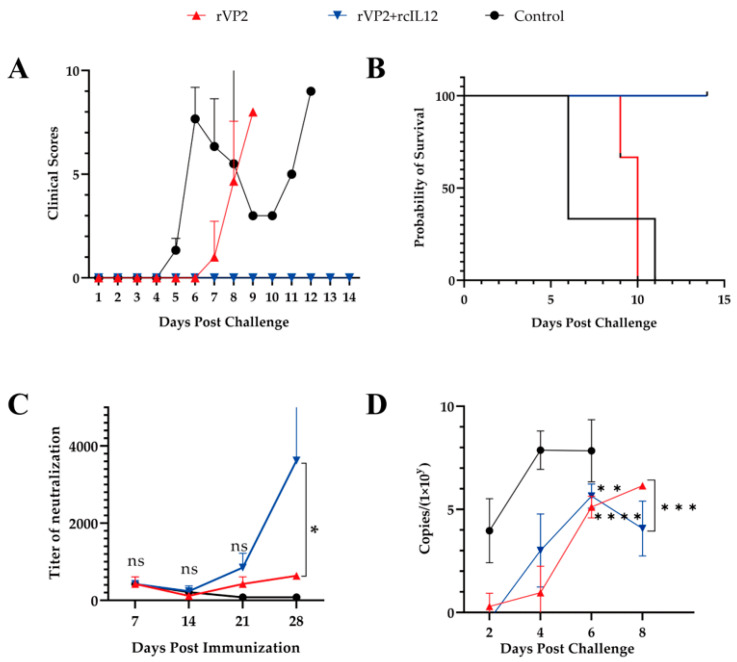
Evaluation of rVP2-IL-12 vaccine efficacy in challenged beagles in rVP2 (red), rVP2+IL-12 (blue) and control (black) groups. (**A**) Clinical scores (0–10 points). (**B**) Survival curves (14-day observation). Mortality: rVP2 (3/3), rVP2+IL-12 (0/3), control (3/3). (**C**) Neutralizing antibody titers at day 7 post-vaccination interval. (**D**) Virus shedding (qPCR, copies/μL) days 1–8 post-challenge. Data represents mean ± S.D. (*n* = 3). * indicates *p* < 0.05, ** indicates *p* < 0.01, ns indicates no significance, *** indicates *p* < 0.001, and **** indicates *p* < 0.0001.

Vaccination was well-tolerated (no adverse events). Post-challenge clinical symptoms were scored according to established criteria (Table 1). Post-challenge, controls developed severe symptoms by Day 4, whereas rVP2 dogs displayed attenuated progression (Day 6 onset). The rVP2+rcIL-12 group showed complete clinical protection (0% morbidity; Figure 3A). Survival rates diverged significantly: rVP2+rcIL-12 (100% survival) vs. rVP2 (median survival: 9 days) vs. controls (6 days) (Figure 3B).

Neutralizing antibody kinetics revealed initial titer reduction post-prime, with rcIL-12 co-administration accelerating secondary responses. By day 28, the rVP2+rcIL-12 group achieved significantly higher titers, demonstrating rcIL-12’s Immune-enhancing potency (Figure 3C).

Viral shedding dynamics were assessed via qPCR of rectal swab-derived DNA. Control dogs shed 10^4^ CPV copies by day 2, peaking at 10^8^ copies by day 4 until death (Figure 3D). In contrast, both vaccinated groups (rVP2 and rVP2+rcIL-12) showed delayed and reduced shedding (undetectable on day 2), reaching 10^6^ copies by day 6. Intergroup analysis (day 6) revealed no significant difference between vaccine groups (*p* > 0.05), but both demonstrated markedly lower shedding versus controls (*p* < 0.01; Figure 3D).

In summary, while rVP2 delayed disease progression and reduced viral shedding (suggesting partial viral blockade) [19], it failed to prevent mortality. In contrast, the rVP2+rcIL-12 regimen induced robust protective efficiency, achieving sterilizing protection (no clinical signs) and 100% survival. This underscores rcIL-12’s critical role in immune-enhancing.

### 3.4. Histopathological Analysis

Necropsy was performed to observe pathological changes in tissues and organs. In the rVP2+rcIL-12 group, all organs appeared normal without lesions. The rVP2 group showed reduced pathological severity in the intestines, liver, spleen, and lungs, while the control group exhibited extensive hemorrhagic lesions.

**Figure 4:**
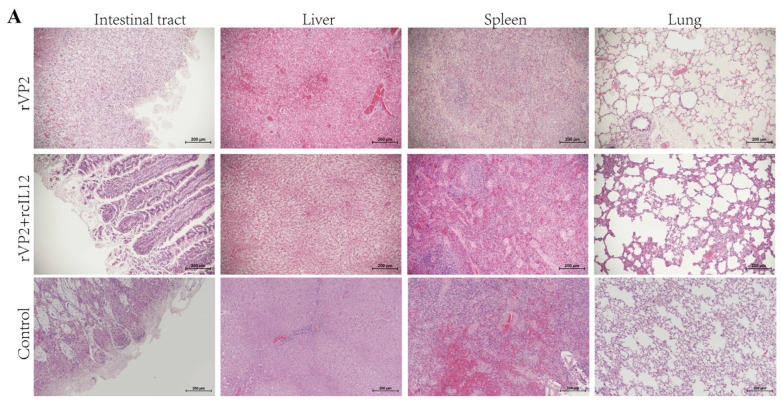

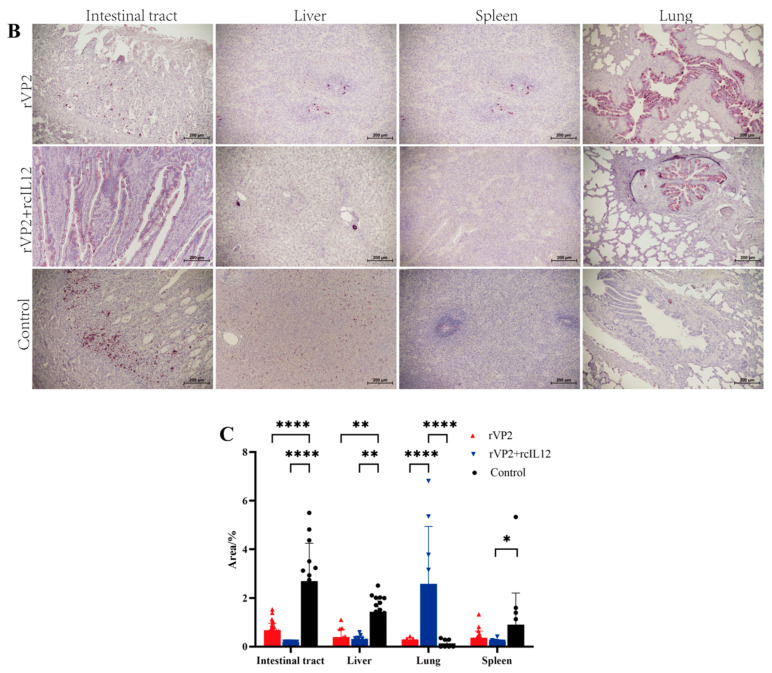
Histopathological and immunohistochemical analysis. (**A**) HE staining. Representative images show, e.g., inflammatory infiltration, necrosis, or tissue structure. Scale bar: 200 µm. (**B**) IHC staining for CPV. Red-brown HRP signals indicate CPV positioning propagation. Scale bar: 200 µm. (**C**) Quantitative analysis of IHC signals. CPV positive cells were counted in random fields per sample and analyzed. Data represents mean ± S.D. (*n* ≥ 5). * indicates *p* < 0.05, ** indicates *p* < 0.01 and **** indicates *p* < 0.0001.

Histopathological examination using HE staining (Figure 4A) revealed that the intestinal villi were severely damaged and detached in the control group, with some compensatory hyperplasia forming epithelial-like structures. In contrast, the rVP2+rcIL-12 group exhibited relatively intact villus morphology, although partial epithelial shedding was observed. The rVP2 group showed relatively extensive villous detachment. Granulomatous lesions with mild inflammatory infiltration and localized congestion were observed in the liver tissues of the control group. No granulomatous changes were detected in the livers of the rVP2+rcIL-12 group, while occasional granulomas and mild inflammatory infiltration were present in the rVP2 group. Splenic histology in the control group showed a marked reduction in lymphoid follicles and lymphocyte numbers, indicative of typical lymphoid follicular depletion. Compensatory monocytic lymphocyte proliferation and severe congestion were also observed in some individuals. In contrast, the rVP2+rcIL-12 group maintained an intact splenic lymphoid structure, without inflammatory infiltration or significant hemorrhage. The rVP2 group displayed relatively dispersed lymphoid structures with partial inflammatory infiltration, but no hemorrhagic lesions were noted. Pulmonary tissue in the control group demonstrated interstitial pneumonia, thickened alveolar walls, and minor hemorrhage in some animals. Mild pulmonary lesions were observed in a subset of animals from both the rVP2+rcIL-12 and rVP2 groups; however, the remaining alveolar structures appeared normal, with no evidence of hemorrhage or other pathological alterations.

As shown in Figure 4B, IHC analysis revealed that in the rVP2+rcIL-12 group, some dogs exhibited mild positive staining at the margins of intestinal goblet cells, while others showed negative signals. The rVP2 group displayed scattered positive signals at the base of the intestinal villi, whereas the control group showed abundant signals in the same region and throughout the villi. In the liver, the rVP2+rcIL-12 group showed no parenchymal staining, but strong positivity was observed in the villous epithelium of hepatic arteries. The rVP2 group exhibited scattered positivity in hepatic sinusoids and central lobules, while the control group showed widespread sinusoidal staining. Splenic tissues were negative in the rVP2+rcIL-12 group, showed scattered positivity in the rVP2 group, and had consistent positive staining in the control group. In lung tissue, bronchial villous epithelium showed uniform positivity in the rVP2+rcIL-12 group, partial positivity in the rVP2 group, and only occasional weak signals in the control group.

Representative IHC images from each group were analyzed using ImageJ 1.8.0 with the IHC Profiler plugin to quantify the percentage of positive staining area. Data were processed in GraphPad Prism 9. Positive signal intensity in intestinal, hepatic, and splenic tissues was significantly lower in both the rVP2+rcIL-12 and rVP2 groups compared to the control group (*p* < 0.001, Figure 4C), rVP2+rcIL-12 group was lower than rVP2 group (liver *p* = 0.9714, spleen *p* = 0.8718, intestine *p* = 0.3326). In bronchial regions, the rVP2+rcIL-12 group exhibited significantly higher epithelial positivity than both the rVP2 and control groups (*p* < 0.05).

The results demonstrate that both the rVP2+rcIL-12 combination and rVP2 alone exhibited antiviral effects in intestinal, hepatic and splenic tissues, with the rVP2+rcIL-12 formulation showing superior efficacy compared to rVP2 alone. Notably, significantly stronger CPV-positive signals were detected in pulmonary bronchi and hepatic arteries in the rVP2+rcIL-12 group compared to other groups (*p* < 0.05). We speculate this may reflect the terminal viral shedding phase in these canines, where the virus was being cleared through hepatic arteries into bloodstream and pulmonary bronchi for external excretion.

## 4. Discussion

CPV is a highly prevalent and pathogenic virus that poses a serious threat to canines, particularly puppies under two months of age. Vaccination serves as the most effective measure to control CPV transmission, with MLVs being currently widely used. However, MLVs are significantly limited by maternal antibody interference, as vaccination before 8 weeks of age may block viral replication and lead to inadequate immune responses [3]. Additional concerns include the risk of virulence reversion and post-vaccination viral shedding. Given that the most susceptible population to CPV infection is precisely these young puppies, the development of novel vaccines capable of overcoming maternal antibody interference and the establishment of revised immunization protocols remain crucial. In this study, we administered a combination of rVP2 and rcIL-12 to 20-day-old puppies to evaluate whether this approach could better circumvent maternal antibody interference compared to rVP2 alone. The results demonstrated that the rVP2+rcIL-12 combination induced significantly higher serum neutralizing antibody titers (6–7 times higher) than rVP2 alone by day 28 post-immunization. Moreover, the combination group exhibited superior outcomes in terms of reduced viral shedding, lower tissue viral loads, and attenuated histopathological lesions following challenge, collectively indicating enhanced protective efficacy against CPV infection.

The CPV VP2 contains multiple critical B-cell epitopes within its N-terminal domain and loop structures [15]. These epitopes can effectively induce neutralizing antibody production during CPV infection, making VP2 an essential component in the development of CPV subunit vaccines. However, subunit vaccines often exhibit limitations in immunogenicity and immune persistence, which can be addressed through the use of immunoadjuvants [20]. As a novel cytokine adjuvant, IL-12 plays a pivotal role in bridging innate and adaptive immunity by promoting IFN-γ production, proliferation, and cytotoxic activity of natural killer cells and T lymphocytes [21]. Numerous studies have demonstrated IL-12’s capacity to enhance antigen immunogenicity while maintaining crucial functions in intestinal homeostasis and inflammatory responses [22]. In veterinary vaccine development, various cytokines, including IL-4, IL-12, IL-18, and IL-1β, have been investigated as adjuvants for vaccines against pseudorabies virus (PRV), chicken anemia virus (CAV) and infectious bursal disease virus (IBDV) [23,24,25,26]. Although these cytokines employ different mechanisms of action (e.g., incorporation into recombinant live vaccines, plasmid construction, or direct injection), they consistently enhance both humoral and cellular immune responses. Given that VP2 protein primarily induces humoral immunity, we employed IL-12 as an immunoadjuvant in this study to augment host immune responses to the vaccine antigen and compensate for this limitation, leveraging IL-12’s ability to stimulate cellular immunity.

In this study, we successfully expressed and purified rVP2 and rcIL-12 proteins (purity > 90%) using baculovirus expression system. The biological activities of these proteins were confirmed through hemagglutination assays, canine PBMC proliferation tests, and IFN-γ induction experiments. The purified proteins were then used to immunize puppies to evaluate the immunoenhancing effect of rcIL-12 on rVP2.

The serum neutralizing antibody titers in the rVP2+rcIL-12 group were consistently higher than in the rVP2-alone group, reaching significantly higher levels by day 28 post-immunization. Post-challenge observations showed that puppies in the rVP2+rcIL-12 combination group exhibited no clinical symptoms such as diarrhea, loss of appetite, hematochezia, or elevated body temperature, with a 100% survival rate. In contrast, while the rVP2-alone group showed delayed onset and milder clinical symptoms, it failed to provide complete protection (0% survival rate). Viral shedding analysis revealed no significant difference between the two vaccinated groups during the first 6 days post-challenge (both significantly lower than the control group), but the combination group showed significantly reduced shedding from days 6 to 8 compared to the rVP2-alone group. Gross pathological examination demonstrated extensive hemorrhagic lesions in the intestines and liver, splenic follicular depletion, and pulmonary hemorrhage in control animals. The rVP2 group showed attenuated pathological changes, while the rVP2+rcIL-12 group maintained nearly normal tissue morphology. HE staining further confirmed these findings: the combination group maintained intact intestinal villi structure, showed no hepatic granulomas, preserved splenic lymphoid follicles, and exhibited only mild pneumonia. IHC analysis indicated that both vaccinated groups had significantly lower tissue viral loads than controls, with the rVP2+rcIL-12 group showing superior viral clearance in all examined tissues compared to the rVP2-alone group.

While rVP2 alone provided partial protection in this study, it failed to confer complete protection in puppies, likely due to high levels of maternally derived antibodies at 20 days of age, which may have neutralized the primary immune response. In contrast, the rVP2+rcIL-12 group exhibited no clinical signs or pathological lesions post-challenge and achieved 100% survival. These findings indicate that rcIL-12 significantly enhances the protective efficacy of rVP2 against canine parvovirus infection.

This study has several limitations. Due to a previous outbreak of CPV causing mortality in some dogs, despite thorough disinfection, residual virus in the facility could not be excluded. Therefore, no concurrent control group was established; instead, age-matched, non-immunized dogs from prior experiments with the same challenge dose were used as controls. Additionally, an rcIL12-only group was not included due to practical constraints. Given IL-12’s key role in intestinal inflammation [22], the possibility that anti-IL-12 antibodies in dogs mitigated post-infection inflammation cannot be ruled out. Moreover, direct IL-12 administration is associated with adverse effects such as fever and cytokine storm and has a short half-life, necessitating further investigation of optimal dosing and mode of administration for IL-12 [27]. The timing of immunization also requires optimization to minimize maternal antibody interference, enhance efficacy, reduce vaccine failure, and improve safety.

## 5. Conclusions

rVP2 and rcIL-12 proteins with bioactivity were successfully expressed and purified using the baculovirus-insect expression system. Through comprehensive evaluations, we demonstrated that rcIL-12 significantly enhances the immunogenicity of rVP2, conferring complete protective immunity against CPV challenge.

## Figures and Tables

**Table 1:** The immunization protocol was conducted as follows: The day of immunization was designated as Day 0. rVP2 (about 1 mg/mL) was emulsified with 10% SEPPIC GEL 02 adjuvant, while rcIL-12 (about 900 μg/mL) was administered directly.

Days	rVP2 Group	rVP2+rcIL-12 Group
Day 0	200 μg rVP2 protein	200 μg rVP2 protein, 100 μg rcIL-12 protein
Day 14	200 μg rVP2 protein	200 μg rVP2 protein, 100 μg rcIL-12 protein
Day 39	10^4^ TCID_50_ units of CPV venom were administered by gavage and subcutaneously	
Day 53	Survival dogs are euthanised	

**Table 2:** Clinical scoring criteria for canine parvovirus infection.

Items of Observation	Standard of Scoring	Scores
Mental Status	Normal	0
	Depressed	1
	Recumbent	2
	Death	3
Appetite Status	Normal	0
	Decreased	1
	Hyporexia (persistent ≥ 2d)	2
	Anorexia (persistent ≥ 2d)	3
Fecal Consistency	Normal feces	0
	Watery diarrhea	1
	Persistent watery diarrhea (≥2d)	2
	Blood-tinged feces	3
	Hemorrhagic diarrhea (≥2d)	4

## Data Availability

Data available in a publicly accessible repository.
